# Perioperative Anesthesia and Acute Smell Alterations in Spine Surgery: A “Sniffing Impairment” Influencing Refeeding?

**DOI:** 10.3389/fsurg.2022.785676

**Published:** 2022-03-16

**Authors:** Matteo Briguglio, Tiziano Crespi, Francesco Langella, Patrizia Riso, Marisa Porrini, Laura Scaramuzzo, Roberto Bassani, Marco Brayda-Bruno, Pedro Berjano

**Affiliations:** ^1^IRCCS Orthopedic Institute Galeazzi, Scientific Direction, Milan, Italy; ^2^IRCCS Orthopedic Institute Galeazzi, Intensive Care Unit, Milan, Italy; ^3^IRCCS Orthopedic Institute Galeazzi, GSpine 4, Milan, Italy; ^4^University of Milan, Department of Food, Environmental and Nutritional Sciences, Division of Human Nutrition, Milan, Italy; ^5^IRCCS Orthopedic Institute Galeazzi, Spine Unit 1, Milan, Italy; ^6^IRCCS Orthopedic Institute Galeazzi, Spine Unit 2, Milan, Italy; ^7^IRCCS Orthopedic Institute Galeazzi, Spine Unit 3, Milan, Italy

**Keywords:** smell disorder, anesthesia, inhalation exposures and halogens, fluorinated hydrocarbons, perioperative period and refeeding, critical care, orthopedic procedures, spine

## Abstract

Medications for general anesthesia can cause smell alterations after surgery, with inhalation anesthetics being the most acknowledged drugs. However, spine patients have been poorly studied in past investigations and whether these alterations could influence the refeeding remains unclear. This research aims to observe detectable dysosmias after spine surgery, to explore any amplified affection of halogenates (DESflurane and SEVoflurane) against total intravenous anesthesia (TIVA), and to spot potential repercussions on the refeeding. Fifty patients between 50 and 85 years old were recruited before elective spine procedure and tested for odor acuity and discrimination using the Sniffin' Sticks test. The odor abilities were re-assessed within the first 15 h after surgery together with the monitoring of food intakes. The threshold reduced from 4.92 ± 1.61 to 4.81 ± 1.64 (*p* = 0.237) and the discrimination ability reduced from 10.50 ± 1.83 to 9.52 ± 1.98 (*p* = 0.0005). Anesthetic-specific analysis showed a significant reduction of both threshold (*p* = 0.004) and discrimination (*p* = 0.004) in the SEV group, and a significant reduction of discrimination abilities (*p* = 0.016) in the DES group. No dysosmias were observed in TIVA patients after surgery. Food intakes were lower in the TIVA group compared to both DES (*p* = 0.026) and SEV (*p* = 0.017). The food consumed was not associated with the sniffing impairment but appeared to be inversely associated with the surgical time. These results confirmed the evidence on inhalation anesthetics to cause smell alterations in spine patients. Furthermore, the poor early oral intake after complex procedures suggests that spinal deformity surgery could be a practical challenge to early oral nutrition.

## Introduction

Thousands of spine patients worldwide are daily subjected to a controlled and reversible loss of consciousness with drugs administered by intravenous infusion or inhalation. General anesthesia is advantageous for the surgeon who operates a motionless body, for the anesthesiologist who has full control of the patient's intrinsic physiological mechanisms, and for the patient who has no pain or future reminiscence (from the Greek *anaisthis*í*a*: α, ν- “without” and -αí, σ*θησις* “sensation”). However, some reports suggest that the patient may experience another shortage: a reduction of the sense of smell. Postoperative smell disorders were observed in different surgical populations, and they have been studied in relation to drugs used for general anesthesia, such as the inhaled DESflurane (DES) and SEVoflurane (SEV) or the intravenous anesthetics (TIVA) (1–3). The anesthetic-induced unconsciousness is known to derive from a general disconnection of higher-order brain centers (4), with connectivity networks being required for olfactory processing (5). Inhaled halogenates can nevertheless be the ones mostly affecting the sense of smell because they also collide with the posterodorsal olfactory epithelium of the nasal cavity that houses the odorant receptors (cranial nerve I). Importantly, these sensory neurons play a fundamental role in driving eating behaviors, and subjects with sniffing impairment can decide to alter their diet to compensate for the loss (6). In fact, the smelling of palatable food aromas promotes appetite, liking, and food intake (7, 8), especially in restrained eaters (9). Fasting patients undergoing surgery refrain from eating from the day before, making early oral food after surgery one of the cornerstones of the perioperative nutritional support program in spine surgery (10, 11). Whether the potential sniffing impairment after surgery could affect the refeeding in surgical patients has never been properly explored, with spine patients being scarcely included in past trials on acute anesthesia-derived decays of the sense of smell.

This observational trial aims at clarifying three research questions. (1) The existence of acute (early 15 h) dysosmias after spine surgery. (2) Any amplified affection of halogenates on the sense of smell vs. the subgroup of patients with halogen-free general anesthesia. (3) If the potential decrease in olfactory abilities could have clinical repercussions on the postoperative refeeding (early 15 h) in terms of energy intakes.

## Materials and Methods

### Study Design and Participants

The study was conducted at IRCCS Orthopedic Institute Galeazzi of Milan, Italy. The research was planned as a prospective observational trial of 50 patients recruited from the population undergoing elective spine surgery. The study protocol was drafted in accordance with the Good Clinical Practice and the current revision of the Declaration of Helsinki. The competent Ethics Committee approved the study on April 11 2019 and the trial was registered on the online resource ClinicalTrials.gov (NCT04194788). The eligibility criteria included Caucasian race, male or female gender, age between 50 and 85, elective spine surgery, signature and acceptance of informed consent. Patients with one of the following characteristics were excluded: stage III–IV heart failure, stage III–V renal failure, cancer, neuropsychiatric diseases, smokers, olfactory, or taste disorders of any nature.

All cohort subjects followed the routine anesthesiology care with antibiotics, antiemetic, proton-pump inhibitors, neuromuscular blocker, antipyretics, anti-inflammatory, and analgesics. Patients underwent general anesthesia with endotracheal intubation. The groups with halogens received propofol IV bolus for induction followed by halogens for balanced general anesthesia maintenance while the TIVA group received continuous IV infusion of propofol. In all groups, analgesia was obtained with fentanyl IV bolus before intubation and maintained with remifentanil during surgery. Standard electrocardiography, non-invasive blood pressure monitoring, SatO_2_ monitoring, end-tidal carbon dioxide (ETCO_2_) monitoring, and urine output monitoring were performed. The depth of anesthesia in the TIVA group was controlled with a brain function monitor (SEDline® of Masimo Corporation, USA) and maintained in the range of 25–50 PSI (Patient State Index) (12). In the DES and SEV groups, anesthesia was maintained to achieve the desirable age-related minimum alveolar end-tidial concentration (MAC) (13). After the surgical procedure, patients were extubated and discharged with Aldrete's score ≥9.

In the first 15 h after surgery, food consumption from in-hospital diets was monitored through bedside examinations, which comprised the first lunch of the day and the first breakfast following surgery. The first meal of the hospital diet included a first course (e.g., pasta in broth), a second course (e.g., cooked ham), vegetables (e.g., boiled carrots), a fruit mousse, and bread at the patient's choice, with a total of 750–900 kcals. The standard breakfast included two rusks, jam, and tea (milk as an alternative), with a total of 100–150 kilocalories (kcal). Condiments during cooking or extra snacks were also considered during the evaluation. The study sample has been subjected to pre- and postoperative assessment of olfaction abilities, being performed within 15 h after surgery before or after the first meal of the day.

### The Sniffing Tests

The olfaction abilities were evaluated by using the threshold and the discrimination tests from the “Sniffin' Sticks” (Burghart Messtechnik GmbH, Tinsdaler Weg 175, 22880 Wedel, Deutchland), which is composed of pen-like devices dispensing odors to evaluate the nasal chemosensory performance. Both tasks generate a score ranging from 1 to 16. Normative data of healthy subjects are available (14), and have been considered as a check of the correct execution of the tests. The two tests were performed according to the instructions for use. Briefly, non-lateralized measures were evaluated presenting a single pen about 2 cm under both patient's nostrils for 2–3 s. A single trained researcher wearing gloves carried out all tests, with the patients not consuming food, chewing, or eating sweets at least 3–4 h before since odor receptors are distinctly more responsive to food aromas in a fed state. The olfaction acuity task determines the olfactory threshold of a subject by using graduated concentrations of *n*-butanol solution (16 triplets of pens, two containing deionized water and the third the odorant). The patient was first familiarized with the pen with the highest concentration. Then, a staircase procedure was started from the most diluted pen, with the patient being asked to identify the odor-containing pen twice in a row (i.e., staircase-reversal trials). The discrimination test evaluates the patient's ability to differentiate odors based on the comparison between three odors (16 triplets of pens, two containing the same non-target odor and the third the target odor). The patient had to choose the pen containing the odor that smells different in each triplet, with no given clue on the correctness of the statements.

### Statistical Analyses

Descriptive statistics have been reported in the form of mean ± standard deviation (min; max) for normally distributed values (Shapiro–Wilk test >0.05) or in the form of median (Q1/Q3) for skewed data. Categorical variables were reported as frequencies or percentages. Sex-differentiation and presbyosmia were analyzed using Pearson correlation as a determinant of the linear association direction and strength. The three research questions have been subsequently investigated using 2-tailed tests. (1) Before-after surgery differences in olfaction abilities of the whole study cohort was investigated using paired sample *t*-test for normally distributed threshold data and Wilcoxon signed-rank test for the skewed discrimination data. (2) The analyses on the variations for each anesthesia group used paired samples *t*-test for normally distributed continuous values (threshold of DES, SEV, TIVA; discrimination of SEV, TIVA) and Wilcoxon signed-rank test for skewed continuous values (discrimination of DES). The variations of olfaction abilities between anesthesia groups have been investigated through the paralleling of delta (Δ) variations using Mann–Whitney *U*-test for DES vs. SEV or TIVA groups, and for SEV vs. TIVA. (3) Food intakes in the first 15 h after surgery have been compared between groups using independent sample *t-*test controlled for the homogeneity of variances (Levene's test > 0.05). The amount of food consumed has been analyzes as the percentage of energy ingested compared to the whole meal presented in the tray of the first breakfast and first lunch. Changes of threshold and discrimination abilities were analyzed against the percentage of food intakes after surgery in the first 15 h. The delta changes were skewed data. Therefore, Spearman correlation was used to observe the existence, strength, and direction of the association. Data analyses were performed by using the Statistical Package for the Social Sciences (SPSS Statistics 22). The locked database to support the findings is available as a Supplementary Material.

## Results

The study cohort comprised 50 consecutive patients (26 females and 24 males). The demographic and clinical characteristics were reported in the following Table 1.

**Table 1:** Demographic and clinical characteristics of the study cohort.

	***n = 50***
Age (years)	65.37 ± 8.13 (50.06; 80.52)
Gender	24 males, 26 females
Weight (kilograms)	73.33 ± 17.44 (40.00; 117.00)
BMI	25.78 ± 4.51 (17.78; 36.75)
CCI	2 (1/3)
ASA	2 (2/2)
**Surgical indication**
Intervertebral disc surgery	16
Spondylolisthesis	16
Lumbar stenosis	10
Deformity	8
**Anesthesia**
TIVA-TCI (halogenates-free)	7
Halogenates (DES/SEV)	43 (36/7)
Induction-extubation (minutes)	123 (81/178)
Aldrete	10 (10/10)

*BMI, body mass index; CCI, Charlson comorbidity index (scores 1–2, mild; scores 3–4, moderate; scores>5, severe); ASA, American society of anesthesiologists physical status classification system (I, healthy; II, mild; III, severe; IV, life threatening; V, moribund; VI, brain-dead); TIVA-TCI, total intravenous anesthesia-target controlled infusion; Aldrete, Aldrete's scoring system (a score≥9 is required for discharge)*.

Sex-differentiation of threshold and discrimination was observed at baseline: 5.00 ± 1.48 and 10.73 ± 1.93 in females vs. 4.83 ± 1.77 and 10.25 ± 1.73 in males. For what concerns baseline presbyosmia, baseline olfaction abilities negatively associated with the years of age (threshold: *r* = −0.385, *p* = 0.006; discrimination: *r* = −0.068, *p* = 0.637). No baseline differences were found between different anesthesia-specific groups for what concerned threshold (DES vs. SEV, *p* = 0.381; DES vs. TIVA, *p* = 0.972; SEV vs. TIVA, *p* = 0.543) or discrimination (DES vs. SEV, *p* = 0.263; DES vs. TIVA, *p* = 0.442; SEV vs. TIVA, *p* = 0.294).

**Figure 1:**
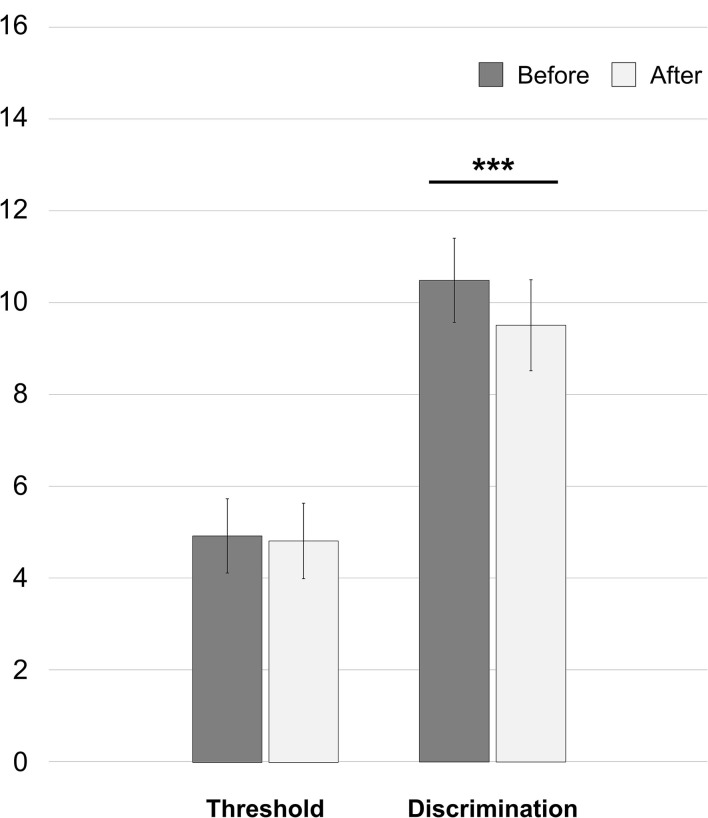
Whole-cohort changes of olfaction abilities in the first 15 h after spine surgery. ***p = *0.0005*.

After spine surgery, the threshold reduced from 4.92 ± 1.61 to 4.81 ± 1.64 [*t*_(49)_ = 1.198; 95% CI: −0.0745 to 0.2945, *p* = 0.237] and the discrimination ability reduced from 10.50 ± 1.83 to 9.52 ± 1.98 (Z = −3.497, *p* = 0.0005). Results are reported in the following Figure 1.

**Figure 2:**
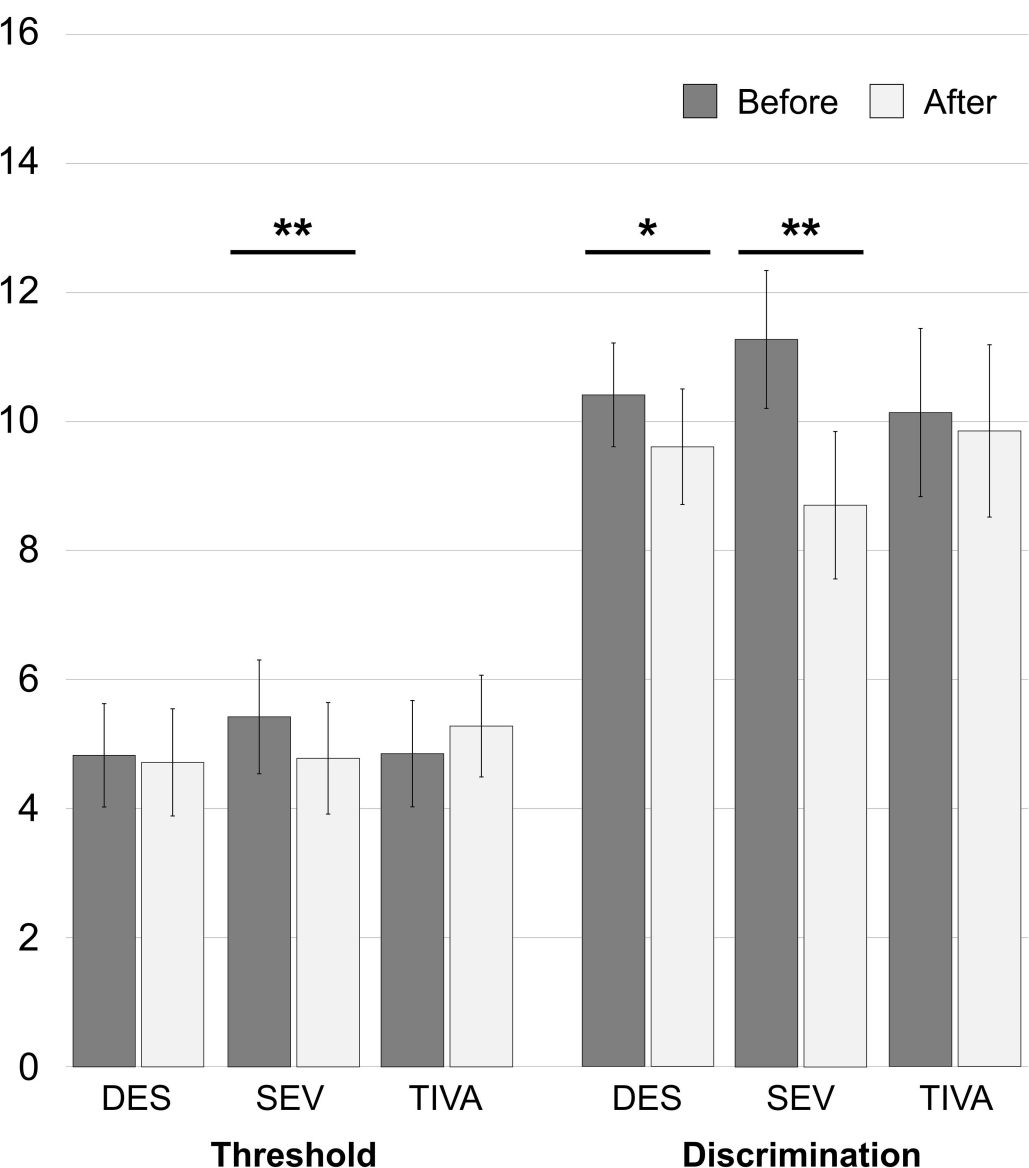
Anesthetic-specific changes of olfaction abilities in the first 15 h after spine surgery. *p = *0.016;* **p = *0.004*.

Patients undergoing anesthesia with halogens experienced a general reduction of threshold from 4.93 ± 1.62 to 4.73 ± 1.65 and of discrimination ability from 10.56 ± 1.71 to 9.47 ± 1.88. Specifically, in the DES group the threshold reduced from 4.83 ± 1.60 to 4.72 ± 1.66 [*t*_(35)_ = 1.160; 95% CI: −0.0833 to 0.3055, *p* = 0.254] and the discrimination reduced from 10.42 ± 1.61 to 9.61 ± 1.79 (Z = −2.403, *p* = 0.016). In the SEV group the threshold reduced from 5.43 ± 1.77 to 4.79 ± 1.73 [*t*_(6)_ = 4.500; 95% CI: 0.2933–0.9924, *p* = 0.004] and the discrimination reduced from 11.29 ± 2.14 to 8.71 ± 2.29 [*t*_(6)_ = 4.500; 95% CI: 1.173–3.970, *p* = 0.004]. In the TIVA group the threshold increased from 4.86 ± 1.65 to 5.29 ± 1.58 [*t*_(6)_ = −1.353; 95% CI: −1.2037 to 0.3465, *p* = 0.225] and the discrimination reduced from 10.14 ± 2.61 to 9.86 ± 2.67 [*t*_(6)_ = 1.000; 95% CI: −0.413 to 0.985, *p* = 0.356]. See Figure 2 for the histogram. The analysis of inter-groups variations showed a difference of both Δthreshold (*U* = 60.500, *p* = 0.025) and Δdiscrimination (*U* = 50.500, *p* = 0.011) between DES and SEV. No difference was observed in Δthreshold (*U* = 80.500, *p* = 0.122) and Δdiscrimination between DES and TIVA (*U* = 110.000, *p* = 0.587). Both Δthreshold (*U* = 4.000, *p* = 0.007) and Δdiscrimination (*U* = 3.000, *p* = 0.005) showed a difference in SEV vs. TIVA.

**Figure 3:**
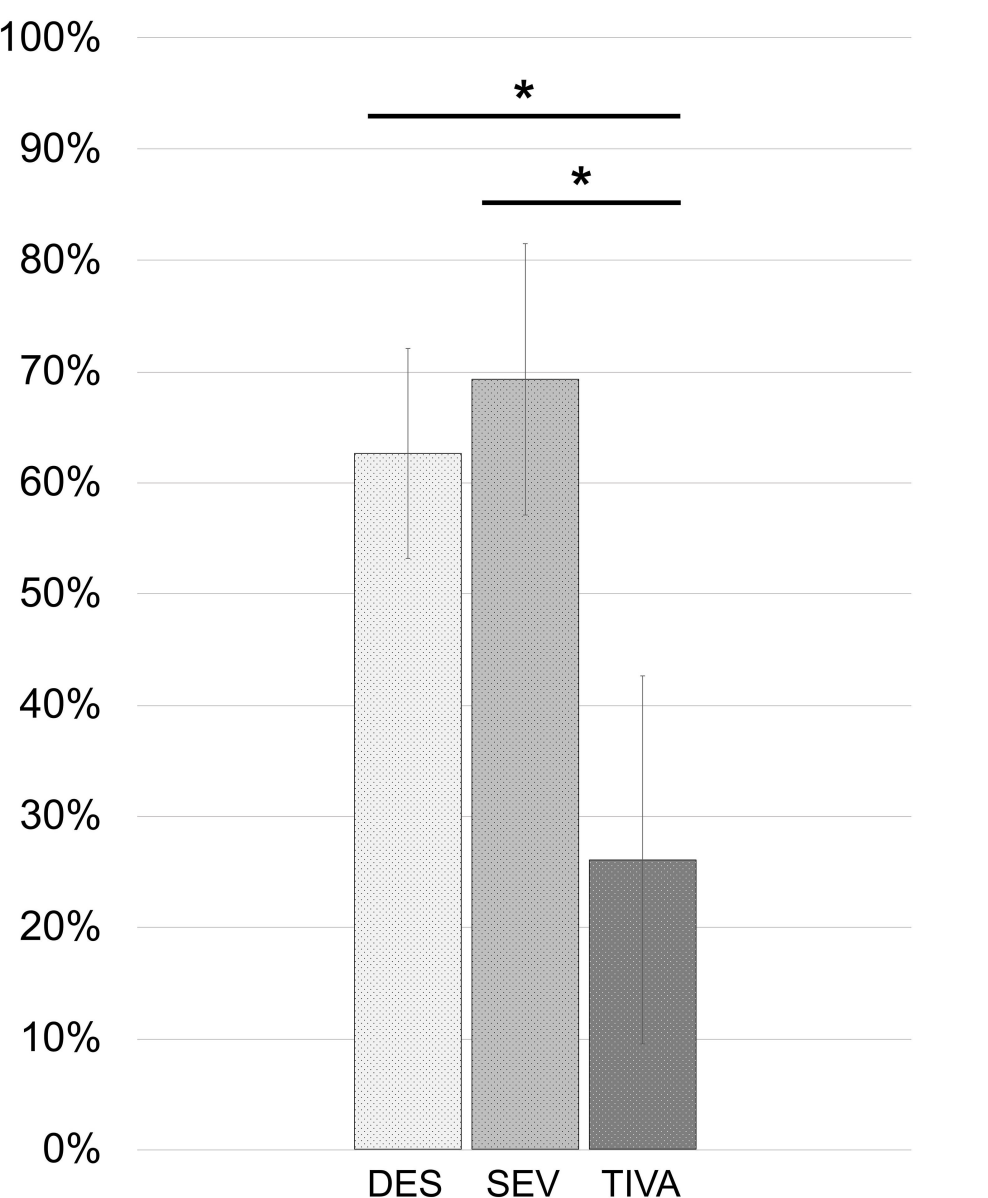
Percentages of food energy ingested from served meals in the first 15 h after spine surgery. *DES vs. TIVA: *p* = 0.026. SEV vs. TIVA: *p* = 0.017.

Concerning the food ingested in the first 15 h, the DES group consumed 84.14 ± 56.77 kcal at breakfast and 486.22 ± 153.01 kcal at lunch. The SEV group consumed 99.57 ± 65.15 at breakfast and 541.43 ± 174.51 kcal at lunch. The TIVA group consumed 7.71 ± 20.41 kcal at breakfast and 239.14 ± 314.47 kcal at lunch. The energy taken from the food was converted into a percentage of the energy of the two meals consumed with respect to the total that was delivered in the tray to the patient's bed in order to decrease any variability in food quality. The percentage of food ingested in the first 15 h after surgery (breakfast plus lunch) in the TIVA group was 26.03 ± 33.11%, which was lower compared to 62.60 ± 18.88% for DES [unequal variance *t*_(6.778)_ = 2.834; 95% CI: −1.2037 to 0.3465, *p* = 0.026) and 69.27 ± 24.43% for the SEV group [equal variance *t*_(12)_ = 2.780; 95% CI: 9.3529–77.1192, *p* = 0.017]. No differences were found between the DES and SEV groups [equal variance *t*_(41)_ = −0.816; 95% CI: −23.1816 to 9.8416, *p* = 0.419]. Results were reported in the following Figure 3. In order to observe a possible association between the reduction of olfaction abilities and the diverse food consumption of the whole cohort, the Δthreshold and Δdiscrimination have been correlated with the percentages of energy intakes. No correlation was found between the percentages of food intakes and Δthreshold [*r*_*s*_(48) = −0.205, *p* = 0.154] or Δdiscrimination [*r*_*s*_(48) = −0.088, *p* = 0.545].

**Figure 4:**
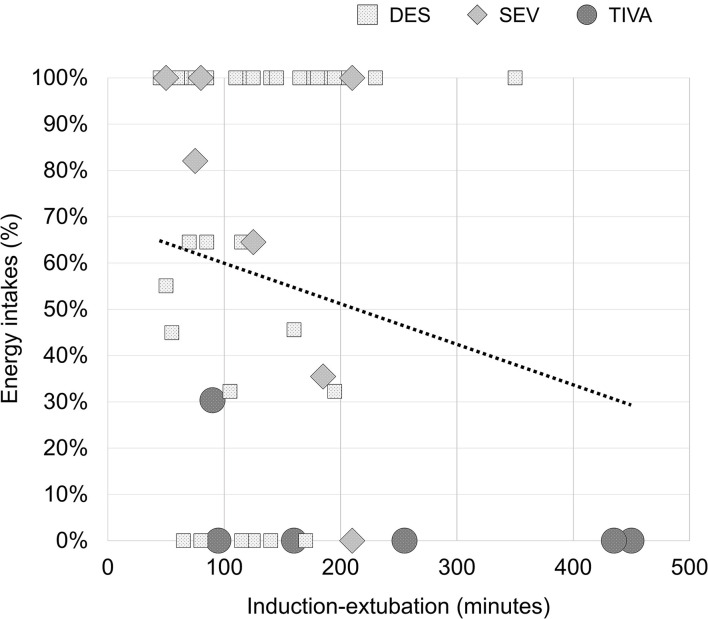
Percentages of early energy intakes after spine surgery depending on the complexity of surgery. *R*^2^ = 0.230 (*p* = 0.002).

Based on the observed absence of association between early food intakes and anesthesia-derived decays, the following *post-hoc* analysis on cofactors influencing food intakes has been conducted. The results of this enquiry are to be considered as hypothesis-generating. Delta changes in threshold and discrimination scores were reduced in a composite factor using the dimension reduction technique of Principal Component Analysis (PCA). DES and SEV groups have been clustered in a single HALO group, and analyzed against the TIVA group. The covariance analysis (ANCOVA) accounted for the following cofactors influencing food intakes: the composite reduction of olfaction abilities, old age (years), comorbid conditions (CCI), perioperative morphine (mg), postoperative numerical rating scale (NRS) for pain (early 15 h), postoperative NRS for nausea (early 15 h), and anesthesia induction-extubation time (minutes) as a measure of surgical complexity. No multiple-model effects have been observed on acute food intakes after controlling for smell affections (*p* = 0.001, adjusted), aging (*p* = 0.0002, adjusted), CCI (*p* = 0.0001, adjusted), morphine (*p* = 0.0004, adjusted), NRS for pain (*p* = 0.0001, adjusted), NRS for nausea (*p* = 0.0002, adjusted). Interestingly, the covariate induction-extubation time did not satisfied the assumption on the variance of the covariate values across the different levels of the independent variable (type of anesthesia) and it could not be included in the ANCOVA. This latter observation drew attention to the complexity of the surgery as a possible obstacle to the proper postoperative refeeding. In fact, The percentages of food intakes correlated with the minutes of induction-extubation [*r*_*s*_(48) = −0.378, *p* = 0.007]. In the next Figure 4, the linear dependence between the two variables was reported (adjusted R Square = 0.230; unstandardized B = −0.144, 95% CI: −0.218 to −0.071; *p* = 0.002). Of note, no association was found between the minutes of induction-extubation and the composite reduction of olfaction abilities [*r*_*s*_(48) = 0.60, *p* = 0.678].

Multiple regression analysis confirmed the predictor potential of the time between anesthesia induction-extubation (unstandardized B = −0.144, 95% CI: −0.243 to −0.045; *p* = 0.005) on the percentage of food intake after surgery, with no contribution observed from smell affections (*p* = 0.117), aging (*p* = 0.725), CCI (*p* = 0.415), morphine (*p* = 0.936), NRS for pain (*p* = 0.904), NRS for nausea (*p* = 0.803).

## Discussion

In this trial, we studied the smell function of spine patients before and after surgery, exploring the different effects on olfaction abilities of general anesthetics and the potential impact on the refeeding. After surgery, the patients of our cohort experienced a significant loss of discrimination ability of 9.33% from baseline. The patients undergoing general anesthesia with SEV encountered an amplified affection on their sense of smell compared to patients receiving DES or halogen-free general anesthesia (TIVA), with a significant postoperative reduction of 11.84% for odor acuity and 22.78% for discrimination from basal scores. Even if patients of the DES group experienced a decrease in discrimination abilities after surgery, the sniffing impairment in the SEV group had been significantly higher than the variations observed in both DES and TIVA patients. Importantly, the observed affections on the sense of smell showed no association with the amount of food consumed after surgery. Manifest differences in terms of early food intakes have been attributed to the complexity of the surgery, meaning the time between anesthesia induction and extubation. Unlike what might have been supposed, advanced age, the presence of comorbidities, the use of morphine, pain, or nausea did not seem to influence the early feeding in our cohort.

Spine surgery is an operation that involves no anatomical locations at potential risk for smell disturbances, and the early onset of sniffing impairments would suggest general anesthetics as a causative factor (15). Olfaction threshold is considered a test assessing dysfunctions at the level of peripheral structures, whereas odor discrimination reflects more the sensineural function of central olfactory processes (16). Odor discrimination testing requires the patient to memorize the suprathreshold smell-containing pens before completing the three-alternative task, and memorizing odors requires, at least to some degree, a differential role of memory. Higher-order brain centers seem to be disconnected from the specificity of the odor stimulus, thus focusing more on hedonic and behavioral values (17). Therefore, we may assume that a peripheral type of dysfunction involved patients of the SEV group whereas a hypo-function of central olfactory processes concerned patients of both halogen groups. The few studies investigating the postoperative effects of general anesthetics on the sense of smell agreed with our results on the superiority of SEV in causing affections of the central olfactory system compared to DES (3) or TIVA (1, 2).

Conversely, to the authors' knowledge this is the first time that early nutrition has been investigated in relation to postoperative sniffing impairment as a mean to contribute with a clinical significance to the research scope. In fact, quantitative olfactory dysfunctions are known to be strongly related to qualitative therefore hedonic misperception of odors (i.e., parosmia) (18), presumably influencing the patients' perceived pleasantness of hospital food (19). In the whole cohort, a considerable portion of the food served was left on the plate, with halogenated and TIVA patients consuming <70 and <30% of the energy served, respectively. Despite the fact that the TIVA patients did not experience any postoperative sniffing impairment, they had been those with the lowest intakes. Regardless of the type of anesthesia, the ingestion of food in the first 15 h has been negatively associated with the length of surgical time, deducing that patients undergoing spinal deformity procedures might be the most at risk of early malnutrition giving that these complex surgeries are usually associated with long operation times. In the subgroup of TIVA patients, in fact, four patients had deformity as primary surgical indication (with two patients having a fusion of 9 or more vertebrae) and represented half of the patients with the same surgical indication in the entire study cohort (see Table 1).

Inhalation of volatile organic compounds has not always occurred for medical purposes. Diethyl ether has long been used for recreational activities from eleventh to the nineteenth century (20). Hydrocarbons in glue, cleaners, or paints were smelled by teenagers who turned on in twentieth-century America, giving birth to the “sniffing syndrome” (21). Glue-sniffing is still widespread in the young population of many countries where any kind of solvent abuse by inhalation is considered an immediate and affordable recreation (22). The *ad libitum* abuse of these ethers is likely to have caused nose irritation (23), contrary to the current halogenated ethers for medical purposes that are known to have a high safety profile and nimbler titratability. It cannot be excluded with certainty a causative role in provoking a mucosal swelling or vasodilation of nasal capillaries that impedes the physical access of odors to the olfactory region, or toxicity damaging of olfactory receptors (24). However, it is reasonable to disregard the possibility of nasal blockage, as the patients would have reported poor nasal breathing. Concerning the existing hypotheses about the pharmacodynamics of general anesthetics, direct interaction with membrane proteins other than indirect lipid bilayer fluidization seem to be the most plausible (25, 26). DES, SEV, and other inhalation anesthetics are known to modulate both synaptic and extrasynaptic GABA_A_ receptors (27, 28), and this implication could substantiate the observed sniffing impairment given the role of GABAergic neuromodulation in olfactory bulb activity (29). Moreover, the anesthesia-derived corruption of higher-order network-level interactions, while leaving local network functions intact (20), could have played a role in disrupting the proper combination of the spatiotemporal pattern of glomerular activation and the corresponding olfactory features, which is necessary for odor discrimination (30). Nonetheless, the loss of consciousness from propofol is also produced by a positive modulation on GABA neurotransmission (31), supporting the prospect either of a propofol interference or of a dissimilar mediator involved in the herein observed sniffing impairment. Of note, individual volatile anesthetics showed some degrees of binding site selectivity in the olfactory epithelium of rats (32).

We can list some limitations of this research. First, the observational nature of the study acquired an uneven allocation of patients between groups, and both SEV and TIVA counted a number of individuals far fewer than the DES group. However, this aspect does not seem to have influenced our research since the current results are arguably similar to those reported by other clinical trials. Second, it cannot be ruled out that the alteration of olfactory discrimination could have been derived from a generalized postoperative cognitive dysfunction (1), though no differences regarding the cognitive status appeared to interest patients emerging either from halogen or TIVA anesthesia (33). Third, the interference from drugs other than morphine in causing the observed effects has not been investigated. For instance, some intraoperative non-steroidal anti-inflammatory drugs may have interfered with the trigeminal activation (34), whose proper *sensitivity* is known to be part of the dynamic interaction with the olfactory system that underlies the perception (35). Besides, the continuous IV infusion of propofol in the TIVA group could have accounted for the difference in non-nutritional calorie burdens that are known to derive from the lipid content of refined soybean oil and purified egg phosphatide (36), thus possibly playing a role in the observed postoperative low food intakes of TIVA patients.

Future studies addressing the anesthesia-derived decays of the sense of smell should consider the use of non-invasive recordings from the olfactory bulb able to detect altered signals and avoid odor habituation, like the electrobulbogram (37). Moreover, there should be the inclusion of tests assessing qualitative olfactory perception, such as the Sniffin' sticks parosmia test (18), in order to observe changes in the odor valence. In conclusion, our study reinforces the evidence on inhalation anesthetics to cause a sniffing impairment after spine surgery. Furthermore, the complexity of these procedures that preclude the prospect of early mobilization to maintain the ideal alignment of the spine could represent a practical challenge also to early oral nutrition. A prudent integration with dietary supplements should be considered to compensate for the lack of nutrition until the complete recovery of the ability to feed on the in-hospital diets (38, 39).

## Data Availability Statement

The original contributions presented in the study are included in the article/Supplementary Material, further inquiries can be directed to the corresponding author.

## Ethics Statement

The studies involving human participants were reviewed and approved by the Ethics Committee of IRCCS San Raffaele Hospital. The patients/participants provided their written informed consent to participate in this study.

## Author Contributions

MB formulated the conception and design of the work, analyzed and interpreted the patient data, and wrote the first draft of the manuscript. MB and TC contributed to the acquisition of data. TC, FL, PR, MP, LS, RB, MB-B, and PB substantively revised the first draft of the manuscript. All authors read and approved the final manuscript. All authors have agreed to be personally accountable for the author's own contributions and to ensure that questions related to the accuracy or integrity of any part of the work, even ones in which the author was not personally involved, are appropriately investigated, resolved, and the resolution documented in the literature.

## Funding

This study was part of the project Ricerca Corrente del Ministero della Salute (Italian Ministry of Health).

## Conflict of Interest

The authors declare that the research was conducted in the absence of any commercial or financial relationships that could be construed as a potential conflict of interest.

## Publisher's Note

All claims expressed in this article are solely those of the authors and do not necessarily represent those of their affiliated organizations, or those of the publisher, the editors and the reviewers. Any product that may be evaluated in this article, or claim that may be made by its manufacturer, is not guaranteed or endorsed by the publisher.
